# 

**Published:** 1915-01-09

**Authors:** 


### CONTENTS.

| | PAGE |
|---|---|
| **Medical Students and the War** ... ... ... | 321 |
| **The Rewards of Institutional Life** ... ... | 322 |
| **Hospital and Institutional News** ... ... ... | 323 |

The New Year Honours—The Indian Honours List—The "Military Cross" and the R.A.M.C. —The New Secretary of Salop County Infirmary—The Voluntary Principle in India—Tuberculosis Dispensaries in London—Mental Institutions and the Wounded—The Radium Department at Manchester. Royal Infirmary—The Record of Sir Perceval Nairne's Chairmanship —The Voluntary Hospitals' Brigade—The New Secretary of the Charity Organisation Society—The Late Mr. A. E. Thomas—The Relation of Hospitals to Panel Doctors—London Insurance Committee and Panel Dispensing—A New Use for an Old Police Station—The Scottish Red Cross Tent Hospital at Rouen—Detraining the Wounded in France—The French Hospitals at Dinan—Christmas Extravagance—Panel Practitioners and Insurance Committees—The Open-air Isolation Hospital—Fever Hospital for Soldiers—This Week's Drug Market.

| | PAGE |
|---|---|
| **The Sick and Wounded in War** ... ... ... | 329 |
| International Law and Usage. | |
| **The Hospitals and the Wounded** ... ... | 330 |
| How to Safeguard Civilian Patients. | |
| **A New Method of Tuberculin Treatment** ... | 331 |
| The Deycke-Much Programme in Germany. | |
| **The Action of Chloroform on the Blood** ... | 332 |
| **Bristol General Hospital** ... ... ... ... | 333 |
| The Recent Extension. | |

[See over.]

### CONTENTS—(*continued*).

| | PAGE |
|---|---|
| **Round the World** ... ... ... ... ... ... | 334 |
| Fellow-Workers and the Roll of Honour. | |
| **Honour for a Poor-Law Official** ... ... ... | 335 |
| Sir Duncombe Mann's Record of Work. | |
| **Gifts and Bequests** ... ... ... ... ... ... | 335 |
| **Hospitals and the War** ... ... ... ... | 336 |
| More Wounded Arrive at Cambridge—Cardiff—Glamorgan and Monmouthshire Hospital—Netley: Christmas at the Welsh War Hospital—Sheffield: 3rd Northern General Hospital. | |
| **National Insurance Act Developments** ... | 337 |
| Report of Departmental Committee on Sickness Benefit Claims. | |
| **The Institutional Library** ... ... ... ... ... | 339 |
| Quain Up to Date. | |
| Bacteriology for Nurses. | |
| The General Practitioner and Syphilis. | |
| The Training of a Sanitary Inspector. | |
| An Old Favourite on Nursing. | |
| **The Editor's Letter-Box** ... ... ... ... | 342 |
| The Cost of Christmas Festivities. | |
| **Clark's Fluorescent Screen Bullet Localiser** | 342 |
| **Personalia** ... ... ... ... ... ... ... | 342 |
| **The Institutional Worker** ... ... (Suppl.) | 1 |
| Answers to December Question: Contributions from Out-patients. | |
| A Fixed Charge the Best. | |
| Personal Appeal the Best Way. | |
| Question for January. | |
| Questions Invited. | |
| The Sick and in Need. | |
| Employment and Training. | |
| Miscellaneous. | |

